# CAF-mediated regulation of prostate cancer stem cell stemness via the Wnt/β-catenin and SDF-1/CXCR4 pathways in castration-resistant prostate cancer

**DOI:** 10.3389/fcell.2025.1617200

**Published:** 2025-07-15

**Authors:** Haoran Chen, Zhen Li, Yuan Yue, Xudong Zhu, Jiazheng Wang, Yafei Chen, Yilin Wang, Zhanyang Luo, Hao Liu

**Affiliations:** ^1^ Guang’anmen Hospital, China Academy of Chinese Medical Sciences, Beijing, China; ^2^ College of Traditional Chinese Medicine, Anhui University of Chinese Medicine, Hefei, Anhui, China; ^3^ The Affiliated Hospital of Southwest Medical University, Luzhou, Sichuan, China; ^4^ Graduate School, Beijing University of Chinese Medicine, Beijing, China; ^5^ Shanghai Pudong Hospital, Fudan University Pudong Medical Center, Shanghai, China

**Keywords:** castration-resistant prostate cancer, cancer-associated fibroblast, prostate cancer stem cell, Wnt/β-catenin, SDF-1/CXCR4

## Abstract

**Introduction:**

The role of cancer-associated fibroblasts (CAFs) in the progression, therapeutic resistance, and metastasis of castration-resistant prostate cancer (CRPC) remains incompletely understood. This study aimed to investigate how CAFs regulate the stemness of prostate cancer stem cells (PCSCs), with a focus on the Wnt/β-catenin and SDF-1/CXCR4 signaling pathways.

**Methods:**

We assessed the expression of CAF and PCSC markers in xenograft tumor tissues from hormone-sensitive prostate cancer and CRPC mouse models using immunohistochemistry and immunofluorescence. The impact of CAFs on stemness markers, SDF-1, CXCR4, and Wnt pathway activation was evaluated both *in vitro* and *in vivo*.

**Results:**

The expression levels of CAF and PCSC markers were significantly elevated in CRPC tissues compared to hormone-sensitive tumors. Bioinformatics analysis indicated high expression of CXCR4 and CTNNB1 (β-catenin) in CRPC, with positive correlations to disease progression. CAFs enhanced PCSC stemness, while inhibition of Wnt3a or SDF-1 led to reduced stemness and pathway activity. *In vivo*, CAFs promoted CRPC tumor growth and significantly increased the expression of Wnt3a, β-catenin, TCF4, LEF1, SDF-1, and CXCR4, along with an elevated p-GSK-3β/GSK-3β ratio. Conversely, β-catenin and CXCR4 inhibitors suppressed tumor growth and downregulated Wnt signaling components.

**Discussion:**

β-Catenin and CXCR4 showed strong co-localization in xenograft tumors. These findings suggest that CAFs promote PCSC stemness and CRPC progression by activating the Wnt/β-catenin and SDF-1/CXCR4 pathways via Wnt3a and SDF-1 expression. These insights provide potential targets for managing CRPC.

## 1 Introduction

Prostate cancer (PCa) is the most common malignant tumor affecting the male reproductive system. In 2022, there were approximately 1.5 million new PCa cases globally, with 397,000 associated deaths (Bray et al., 2024). Recent data indicate an increase in the incidence of PCa, especially among young individuals. A 2024 National Oncology Survey report showed that PCa is the second leading cause of cancer-related deaths among men in the United States (Siegel et al., 2024). This malignancy poses a substantial threat to the health of men, highlighting the urgent need for effective therapeutic strategies.

Recent findings suggest that prolonged PCa treatment may result in varied clonal selection (Cheng et al., 2022) and acquisition of stem-like features (Verma et al., 2020), eventually contributing to the emergence of castration-resistant PCa (CRPC). CRPC represents the refractory stage of PCa, where the tumor re-progresses and develops resistance following castration surgery or anti-androgen therapy, resulting in a poor prognosis, with a 5-year survival rate of approximately 30% (Tang et al., 2022). Tumor heterogeneity, drug resistance, recurrence, and limited therapeutic options complicate CRPC treatment. A study classifying CRPC subtypes using a transposase-accessible chromatin assay along with sequencing (Mu et al., 2024) revealed that androgen receptor (AR)-low/negative subtypes were mostly stem cell-like and Wnt-dependent, whereas transcriptome analysis showed that stem cell-like subtypes were the second most prevalent CRPC subtypes after AR-positive subtypes. These findings highlight the heterogeneity of CRPC and indicate that different subtypes may utilize distinct signaling pathways to evade treatment effect. Additionally, PCa stem cells (PCSCs) likely contribute considerably to the heterogeneity and therapeutic resistance observed in CRPC (Menssouri et al., 2023).

PCSCs, a distinct subpopulation of cells residing within the prostate “niche” (Sun et al., 2024), exhibit self-renewal and multi-lineage differentiation potentials. Notably, these cells exhibit intrinsic resistance to the cytotoxic effects of endocrine therapies such as abiraterone and enzalutamide (Vlashi and Pajonk, 2015), as well as chemotherapeutic agents such as docetaxel (Koukourakis et al., 2023). Additionally, PCSCs can adapt and evolve in response to selective treatment pressures by employing mechanisms such as enhanced expulsion of drugs and activation of anti-apoptotic and DNA repair mechanisms (Chen C. Y. et al., 2024). CD133 and CD44 are specific markers of stem cell-like properties in various cancer types; they have been widely studied in PCa. Research has shown that these markers aid in the isolation of PCSCs that show high drug resistance and strong tumorigenic potential (Fang et al., 2024).

PCSCs rely on the tumor microenvironment (TME) for survival and the maintenance of stem-like properties. Cancer-associated fibroblasts (CAFs), essential elements of the TME, facilitate the survival, proliferation, and self-renewal of PCSCs by secreting growth factors and cytokines, and remodeling the extracellular matrix (Hou et al., 2024). Recent research has indicated that CAFs have the potential to augment cancer stem cell (CSC) activity and promote tumor growth by activating the Wnt/β-catenin and human stromal cell-derived factor-1 (SDF-1)/C-X-C motif chemokine receptor 4 (CXCR4) signaling pathways (Chen H. et al., 2024). The Wnt/β-catenin pathway, a crucial regulatory mechanism for PCSCs, enhances DNA repair capacity and reduces apoptosis, thereby sustaining the stemness and self-renewal ability of PCSCs (Li et al., 2013). Additionally, SDF-1 secreted by CAFs binds to CXCR4, further activating downstream signaling pathways that enhance the stemness and invasiveness of CSCs (Stuart et al., 2019). However, while the independent roles of these pathways have been previously studied, only a few studies have systematically investigated their potential synergistic effects on PCSC stemness in the context of CRPC. To our knowledge, this the first study to elucidate how CAFs may simultaneously coordinate Wnt/β-catenin and SDF-1/CXCR4 signaling to sustain PCSC activity and contribute to CRPC progression. By focusing on this dual-pathway interaction, our preclinical research uncovers a novel mechanism underlying therapeutic resistance and offers new potential targets for the development of combination therapies for CRPC.

## 2 Materials and methods

### 2.1 Reagents and antibodies

Cell culture media and supplements, including RPMI 1640, DMEM, and fetal bovine serum (FBS), were purchased from Life Technologies (Thermo Fisher Scientific, Waltham, MA, United States). The details of the reagents and antibodies are presented in Supplementary Table S1.

### 2.2 Cell culture

Hormone-sensitive prostate cancer cells (LNCaP cells, RRID: CVCL_0395) were procured from Servicebio (Wuhan, China), castration-resistant prostate cancer cells (C4-2B cells, RRID: CVCL_4784) from BeNA Culture Collection (Henan, China), and human prostate stromal myofibroblast cell line (WPMY-1 cells, RRID: CVCL_3814) from Gain Biological (Shanghai, China). Each cell line was cultured in RPMI 1640 medium supplemented with 10% FBS, 100 U/mL penicillin, and 100 μg/mL streptomycin (Sigma–Aldrich, St. Louis, MO, United States). The cells were incubated at 37°C in an atmosphere containing 5% CO_2_. All cell lines used in this study were authenticated via short tandem repeat (STR) profiling within the past 3 years. Furthermore, all experiments were conducted using *Mycoplasma*-free cells to ensure the accuracy and reliability of the data.

### 2.3 Induction and identification of CAFs

CAF formation was induced using TGF-β1, as previously described (Zhang et al., 2020). Briefly, WPMY-1 cells were seeded in a six-well plate at a density of 5 × 10^4^ cells/well. After 48 h of incubation, the medium was discarded and the cells were incubated in complete medium containing TGF-β1 at varying concentrations (0, 10, and 20 ng/mL). Cell morphology was observed after 48 h of TGF-β1 treatment using an optical microscope (BX51; OLYMPUS, Tokyo, Japan). Additionally, the identity of the induced CAFs was confirmed by assessing the expression of fibroblast activation protein (FAP) and α-smooth muscle actin (α-SMA) using immunofluorescence and Western blot assays.

### 2.4 Preparation of culture medium for CAFs

CAFs were treated with the Wnt inhibitor DKK-1 (20 ng/mL) or an SDF-1 neutralizing antibody (1000 nmol/L) for 24 h. WPMY-1 cells, along with untreated and treated CAFs, were seeded separately in six-well plates at a density of 1.5 × 10^5^ cells/mL and cultured for 72 h. After incubation, the medium was collected and centrifuged at 300 *g* for 5 min at room temperature to remove cellular debris. The resulting supernatants were collected as conditioned media (CM) for further analysis: WPMY-1-CM, CAF-CM, CAF^anti−Wnt^-CM, and CAF^anti−SDF-1^-CM.

### 2.5 Enzyme-linked immunosorbent assay (ELISA)

The aforementioned CAF-CM and WPMY-1-CM were collected, and Wnt3a and SDF-1 levels were analyzed using ELISA. Detailed experimental procedures can be found in the Supplementary Methods.

### 2.6 Flow cytometry sorting and sphere formation assay

C4-2B cells were washed 1–2 times with PBS containing 5% FBS (staining solution). To the staining solution (50 μL), 1–2 μL of anti-human Fc blocking antibody (1:100, eBioscience, San Diego, CA, United States) was added, and the cells were incubated at 4°C for 30 min. Next, 2 μL of APC anti-human CD133 antibody (1:100; Biolegend, San Diego, CA, United States) and 2 μL of PE anti-human CD44 antibody (1:100; Biolegend) or isotype control antibodies (1:100; Biolegend) were added to the staining solution (50 μL), and the samples were incubated at 4°C for 30 min. After incubation, the cells were washed twice with PBS. The cell concentration was adjusted to 1 × 10^6^/mL, and the CD133^+^CD44^+^ C4-2 cells were sorted using a flow cytometer (FACS Aria III; BD Biosciences, Franklin Lakes, NJ, United States). The sorted CD133^+^CD44^+^ cells were seeded in a serum-free tumor sphere culture medium supplemented with EGF and bFGF, and cultured in suspension on low-attachment plates. The cells were treated with WPMY-1-CM or CAF-CM for 24 h, and treatments were repeated every 3 days. After 14 days, sphere formation was observed under a microscope (BX51; OLYMPUS). To assess stemness and self-renewal capacity, the sphere formation rate was calculated based on the number of cell aggregates with a diameter greater than 50 μm and a compact, rounded morphology.

### 2.7 Indirect CAF and PCSC Co-Culture model

PCSCs were seeded in six-well plates at a density of 5 × 10^4^ cells per well. CAFs were cultured separately on the 0.4-μm polyester membrane of a 12-mm Transwell insert (Corning, New York, NY, United States). After 24 h, the Transwell inserts were transferred into the wells containing PCSCs to establish an indirect co-culture system. PCSCs and CAFs were co-cultured at ratios of 1:3, 1:6, and 1:9. Cell proliferation was evaluated using the CCK-8 assay to determine the optimal ratio for subsequent experiments.

### 2.8 TOPFlash/FOPFlash luciferase reporter assay

TOPFlash and FOPFlash luciferase activities were measured using a dual-luciferase reporter assay to assess the activity of the Wnt/β-catenin signaling pathway after the treatment of PCSCs with CAF-CM and CAF^anti−SDF-1^-CM. Detailed experimental procedures are provided in the Supplementary Methods.

### 2.9 Animals and treatments

Male NSG mice (4 weeks old, average weight 20 ± 3 g) were obtained from Vital River (Beijing, China). To establish a subcutaneous tumor model mimicking the tumor microenvironment, prostate cancer stem cells (PCSCs, 1 × 10^6^) were injected alone or in combination with cancer-associated fibroblasts (CAFs, 6 × 10^6^) at a 1:6 ratio into the right flank of each mouse in 100 μL of PBS/Matrigel (1:1). This co-injection model was designed to reflect the stromal support provided by CAFs *in vivo*. The mice were randomly divided into four groups (n = 4 per group). Group A received PCSCs alone. Groups B–D received PCSC + CAFs. Once tumor volumes reached approximately 50 mm^3^, the mice were treated as follows: (a) PCSC alone + saline; (b) PCSC + CAFs + saline; (c) PCSC + CAFs + XAV939 (10 mg/kg, i.p.; MedChemExpress, Monmouth Junction, NJ, United States); and (d) PCSC + CAFs + AMD3100 (5 mg/kg, i.p.; MedChemExpress). XAV939 and AMD3100 were administered daily for 21 consecutive days. The drug concentrations were selected based on previously published studies and validated through preliminary experiments for determining dose range to ensure tolerability and efficacy. After 21 days of treatment, the mice were anesthetized with pentobarbital sodium (80 mg/kg, i.p.) and euthanized. Tumors were excised and measured; their volumes were calculated as V = 0.5 × H^2^ × L, where H is the shorter diameter and L is the longer diameter. All animal procedures were approved by the Ethics Committee of Guang’anmen Hospital (Approval No.: IACUC-GAMH-2024-058-SQ) and conducted in accordance with institutional and national guidelines.

### 2.10 Immunofluorescence and immunohistochemical assays

Immunofluorescence was used to determine Wnt, β-catenin, SDF-1, CXCR4, and p-GSK-3β expression in excised tumor tissues, and immunohistochemistry was employed to assess the expression of FAP and α-SMA in tumor tissues. Detailed experimental steps can be found in the Supplementary Methods.

### 2.11 Bioinformatic analysis

Publicly available single-cell RNA sequencing data (GSE193337) (Song et al., 2015) and transcriptome sequencing data (GSE32269) (Zhao et al., 2014) were obtained from the Gene Expression Omnibus database. The GSE193337 dataset contains data of human tumor and benign prostate adenocarcinoma samples, including both single-cell and bulk RNA sequencing data. The GSE32269 dataset contains gene expression data of primary localized and castration-resistant bone metastatic PCa samples. For the GSE193337 dataset, analysis was performed using the Seurat package (Lu et al., 2015). The analysis steps included quality control (removal of low-quality cells and genes), dimensionality reduction (principal component analysis and *t*-SNE), cell clustering, and differential gene expression analysis. For the GSE32269 dataset, differential expression analysis was conducted using the edgeR software package (version 4.0.2). First, the raw data were normalized, followed by quantitative analysis to identify genes with significant expression differences between groups. mRNA expression data for correlation analysis were obtained from the UCSC XENA database. The data were converted into transcripts per million format and standardized using log_2_ transformation. To preprocess raw RNA-seq count data (GSE32269), low-quality reads were removed, gene expression was calculated, and a count matrix was generated. These preprocessing steps were performed using STAR software for genome alignment and featureCounts for counting reads mapped to genes. The final count matrix was used as input for differential expression analysis.

### 2.12 RNA isolation and reverse transcription quantitative real-time PCR (RT-qPCR)

Tumor tissues were sectioned and promptly cryopreserved in liquid nitrogen. Briefly, tissue samples were pulverized into powder using a tissue grinder, followed by RNA extraction using an adequate quantity of TRIzol reagent. cDNA was generated from the extracted RNA using reverse transcriptase. Thereafter, RT-qPCR was performed using ABI 7500 RT-PCR equipment (Thermo Fisher Scientific) with specific reagents and primers. The PCR sequences are listed in Supplementary Table S2.

### 2.13 Western blot analysis

Proteins were extracted on ice using RIPA lysis buffer containing protease inhibitors, and protein concentration was measured using a BCA protein assay kit (Thermo Fisher Scientific). Protein samples (20–50 μg) were separated using SDS-PAGE and transferred onto PVDF membranes. The membranes were blocked with 5% non-fat milk for 1 h and incubated overnight with primary antibody (1:1000) at 4°C, and then with secondary antibody (1:2000) for 1 h at 20°C–25°C. Immunoreactive bands were detected using a ChemiDoc XRS + chemiluminescence detection system (Bio-Rad, Hercules, CA, United States) and analyzed using ImageJ software. Protein expression was normalized to the expression of the internal control GAPDH.

### 2.14 Statistical analysis

Statistical analysis was performed using GraphPad Prism 9 (GraphPad Software, San Diego, CA, United States). All data are presented as mean ± standard deviation from at least three independent experiments. For comparisons among multiple groups, one-way analysis of variance (ANOVA) was conducted, followed by appropriate *post hoc* tests, including Tukey’s, Sidak’s, and Dunnett’s multiple comparison tests. For comparisons between groups, independent sample *t*-tests were used. Results with p-value <0.05 were considered statistically significant.

## 3 Results

### 3.1 High expression of CAF and PCSC markers in CRPC

**FIGURE 1:**
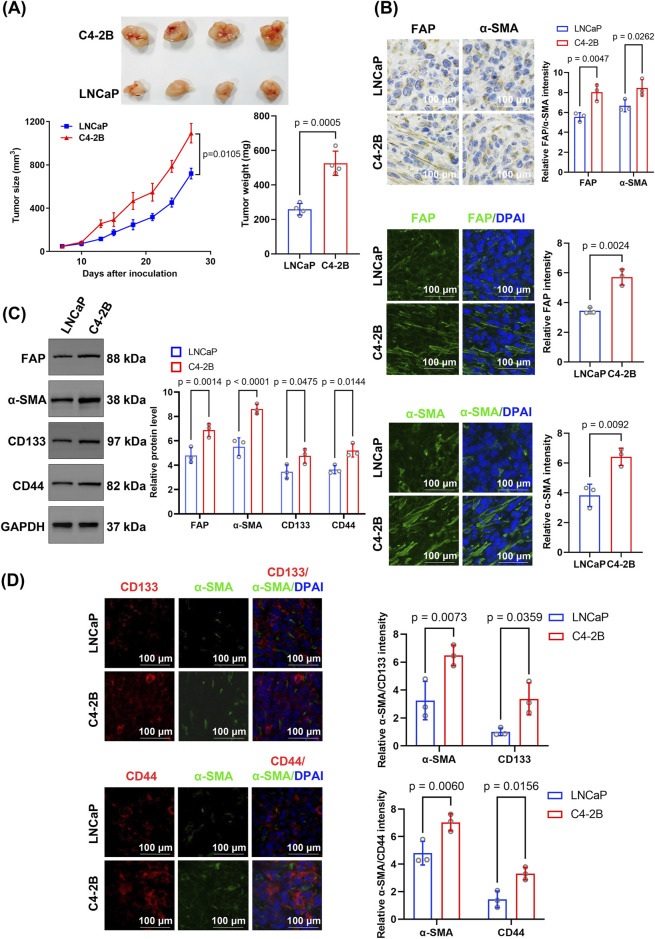
Comparative analysis of tumor growth, stromal activation, and cancer stem cell marker expression between C4-2B and LNCaP xenografts. **(A)** Tumor growth and weight comparison. Tumor growth curves were analyzed using repeated-measures ANOVA, and final tumor weights were compared using unpaired two-tailed Student’s t-test (n = 4 biological replicates per group). **(B)** Immunohistochemical and immunofluorescence analyses of the stromal activation markers FAP and α-SMA. FAP and α-SMA levels were higher in C4-2B than in LNCaP tumors. Quantification was based on average fluorescence intensity per mouse, and statistical analysis was performed using unpaired two-tailed *t*-test (n = 4 biological replicates). **(C)** Western blotting indicated significantly higher levels of FAP, α-SMA, CD133, and CD44 in C4-2B than in LNCaP tumors. Blots were derived from three independent protein extractions and quantified using densitometry normalized to GAPDH expression (n = 3 technical replicates); statistical comparisons were performed using an unpaired two-tailed *t*-test. **(D)** Dual immunofluorescence staining for α-SMA and CD133, or CD44. C4-2B tumors displayed significantly higher fluorescence intensities for CD133 and CD44 than LNCaP tumors. Quantification was based on per-mouse averages and analyzed using an unpaired two-tailed *t*-test (n = 4 biological replicates). Data are presented as mean ± SD.

To investigate the role of CAFs in PCSC stemness regulation during CRPC progression, we established mouse models bearing hormone-sensitive prostate cancer (HSPC) (LNCaP) or CRPC cells (C4-2B). *In vivo*, C4-2B tumors grew significantly faster and larger (*p* = 0.0105) than LNCaP tumors; C4-2B and LNCaP tumor volumes reached 1092.78 ± 154.71 and 720.00 ± 84.49 mm^3^, respectively, by 27 days post-inoculation (Figure 1A).

Further immunohistochemical analysis showed that the CAF markers FAP and α-SMA exhibited significantly higher expression in C4-2B tumor tissues than in LNCaP tumors (FAP: *p* = 0.0047, α-SMA: *p* = 0.0262) (Figure 1B). Similarly, immunofluorescence staining confirmed that the expression of FAP (*p* = 0.0024) and α-SMA (*p* = 0.0092) was significantly higher in C4-2B than in LNCaP tumors (Figure 1B). Additionally, Western blotting showed that FAP (6.88 ± 0.45 vs. 4.80 ± 0.53; *p* = 0.0014) and α-SMA (8.61 ± 0.33 vs. 5.52 ± 0.93; *p* < 0.0001) protein levels were significantly higher in C4-2B than in LNCaP tumors (Figure 1C). Moreover, the expression of the CSC markers CD133 (4.76 ± 0.49 vs. 3.46 ± 0.47; *p* = 0.0475) and CD44 (5.20 ± 0.44 vs. 3.64 ± 0.28; *p* = 0.0144) was significantly higher in C4-2B than in LNCaP tumors (Figure 1C).

Notably, α-SMA expression and the relative fluorescence intensities of CD133 (*p* = 0.0359) and CD44 (*p* = 0.0156) were significantly higher in C4-2B than in LNCaP tumors. Additionally, dual staining revealed spatial proximity between the CAF marker α-SMA and tumor stem cell markers CD133 and CD44 (Figure 1D).

### 3.2 Induced transformation of CAFs and their role in maintaining PCSC stemness

**FIGURE 2:**
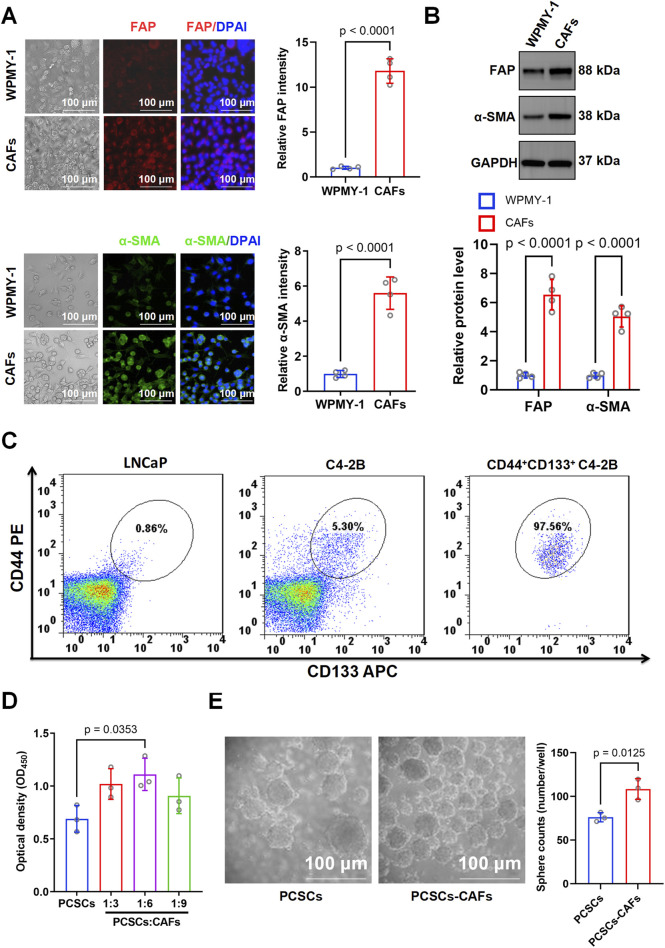
Characterization of cancer-associated fibroblasts (CAFs) and their effects on prostate cancer stem cell (PCSC) proliferation and stemness. **(A)** Immunofluorescence staining for FAP and α-SMA in WPMY-1 cells and CAFs. Representative images show significantly higher expression of FAP and α-SMA in CAFs than in WPMY-1 cells. Quantification was based on three independent experiments, and statistical comparisons were performed using unpaired two-tailed *t*-test (n = 3 technical replicates). **(B)** Western blotting showed that FAP and α-SMA protein levels were significantly higher in CAFs than in WPMY-1 cells. Blots were derived from three independent protein extractions and analyzed using an unpaired two-tailed *t*-test (n = 3 technical replicates). **(C)** Flow cytometry analysis of CD44^+^CD133^+^ subpopulations in LNCaP and C4-2B cells. Compared with LNCaP (0.91%) and unsorted C4-2B cells (5.30%), CD44^+^CD133^+^ cells were highly enriched post-sorting (97.56%). **(D)** Co-culture of PCSCs with CAFs at ratios of 1:3, 1:6, and 1:9. Cell proliferation was assessed based on OD450 measurement using the CCK-8 assay. PCSCs co-cultured with CAFs at a 1:6 ratio showed significantly increased proliferation compared to PCSCs alone. Data were obtained from three independent experiments (n = 3 technical replicates), and analyzed using one-way ANOVA followed by *post hoc* Tukey’s test. **(E)** Sphere formation assay showed that co-culturing with CAFs significantly enhanced the number of spheres formed by PCSCs (108.43 ± 9.66 spheres/well) compared with that in the PCSC-only group (76.13 ± 4.33 spheres/well). Sphere numbers were counted from three independent experiments and analyzed using an unpaired two-tailed *t*-test (n = 3 technical replicates). Data are presented as mean ± SD.

TGF-β1 is known for its essential role in the induction of CAFs (Pan et al., 2024). In this study, WPMY-1 cells were treated with 10 ng/mL TGF-β1, a concentration selected based on optimization experiments using different TGF-β1 concentrations (Supplementary Figure S1). Immunofluorescence staining revealed a marked increase in FAP and α-SMA expression following TGF-β1 treatment compared with that in the untreated group (Figure 2A). Western blotting confirmed the increased expression of FAP (*p* < 0.0001) and α-SMA (*p* < 0.0001) in CAFs, highlighting that the induced cells displayed typical CAF characteristics (Figure 2B). Overall, these results indicate that TGF-β1 successfully induced the transformation of WPMY-1 cells into highly activated CAFs.

The stem cell markers CD44 and CD133 can be used to sort cell subtypes among C4-2B cells (Li and Mu, 2023; Wang et al., 2023). Flow cytometry analysis showed that CD44^+^CD133^+^ prostate cancer stem-like cells accounted for 0.86% of the LNCaP population and 5.30% of the unsorted C4-2B population. After sorting, the proportion of CD44^+^CD133^+^ cells in C4-2B increased to 97.56%, indicating successful enrichment of the stem-like subpopulation (Figure 2C). In the Transwell co-culture system, the CCK8 assay indicated a marked increase in the proliferation rate of PCSCs cultured with CAFs (1:6 ratio) (Figure 2D). To further investigate the effect of CAFs on PCSC stemness, we examined the spheroid formation rate in the PCSC and PCSC–CAF (1:6) groups. Compared with that in the PCSC-only group (76.13 ± 4.33 spheroids/well), spheroid formation increased significantly (*p* = 0.0125) to an average of 108.43 ± 9.66 spheroids/well in the PCSC–CAF co-culture group, underscoring the essential role of CAFs in sustaining PCSC stemness (Figure 2E).

### 3.3 Distribution characteristics of CAFs in PCa and correlation analysis between CXCR4 and the Wnt/β-catenin signaling pathway

**FIGURE 3:**
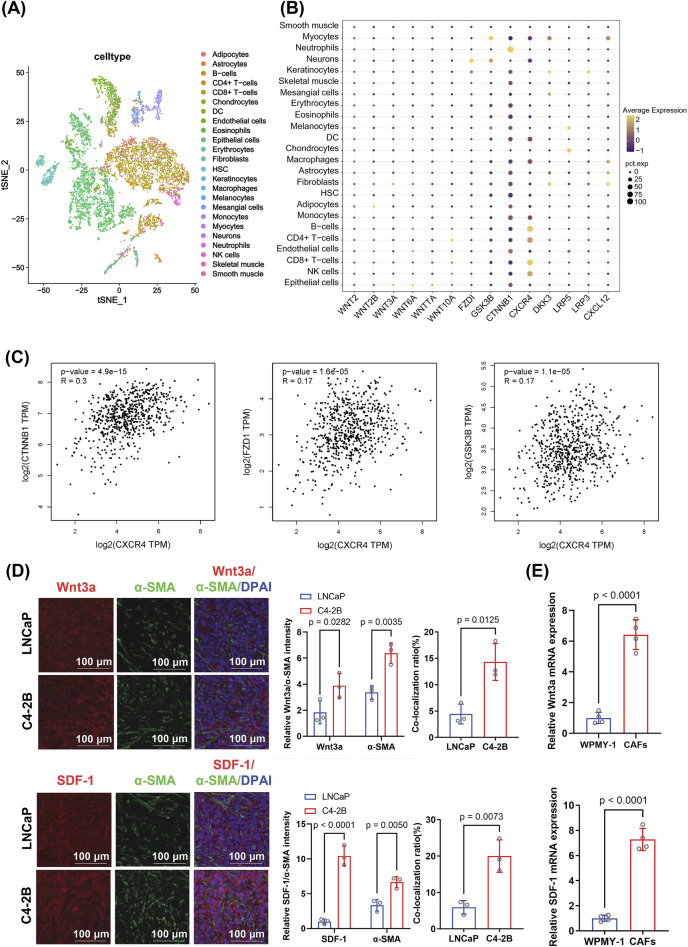
Analysis of expression patterns and correlations of genes involved in the Wnt/β-catenin and SDF-1/CXCR4 pathways in prostate cancer, focusing on their differential expression in CAFs. **(A)** t-SNE clustering showing the distribution of various cell types in the tumor microenvironment. The distinct clustering of fibroblasts suggests a potential regulatory role in the castration-resistant prostate cancer microenvironment. **(B)** Dot plot illustrating elevated expression of genes related to Wnt/β-catenin and SDF-1/CXCR4 signaling in fibroblasts. Dot size represents gene expression level; color reflects statistical significance. Differential expression was assessed using the Wilcoxon rank-sum test. **(C)** Correlation analysis between *CXCR4* expression and key Wnt/β-catenin pathway genes (*CTNNB1*, *FZD1*, and *GSK3-β*). Pearson correlation coefficients and corresponding p-values were calculated. **(D)** Immunofluorescence staining for Wnt3a, SDF-1, and α-SMA in C4-2B and LNCaP tumor tissues. The CAF-associated markers Wnt3a and SDF-1 showed higher expression and co-localization with α-SMA in C4-2B tumors. Quantification was based on fluorescence intensity per mouse and analyzed using an unpaired two-tailed *t*-test (n = 4 biological replicates). **(E)** RT-qPCR analysis of Wnt3a and SDF-1 mRNA levels in CAFs and WPMY-1 cells. mRNA expression was normalized to GAPDH expression and analyzed using an unpaired two-tailed *t*-test from three independent experiments (n = 3 technical replicates). Data are presented as mean ± SD.

To explore the cell-type-specific expression landscape of key signaling molecules in PCa, we performed dimensionality reduction using the t-SNE algorithm based on single-cell transcriptomic data (Figure 3A). This analysis enabled visualization of diverse stromal and immune subpopulations within the PCa microenvironment. Among these, CAFs formed a distinct cluster, prompting further investigation of their molecular characteristics.

A dot plot analysis of key genes related to Wnt signaling and stemness regulation revealed that *CXCR4* and *CTNNB1* (the gene encoding β-catenin) were widely expressed across multiple cell types, including fibroblasts, with particularly high levels in CRPC (Figure 3B). The high variability in expression suggests their dynamic involvement in tumor progression. Furthermore, analysis of TCGA data revealed a statistically significant but weak positive correlation between *CXCR4* and *CTNNB1* expression (*R = 0.3, p < 0.0001*), as well as modest correlations between *CXCR4* and other Wnt-related genes, such as *FZD1* and *GSK-3β* (Figure 3C), suggesting that these molecules may be co-regulated within a shared signaling axis.

Additionally, immunofluorescence staining was performed to investigate whether Wnt3a, SDF-1, and α-SMA are co-localized to C4-2B and LNCaP tumors. In the immunofluorescence co-localization experiment, the expression of Wnt3a and SDF-1 was significantly higher in C4-2B tumors than in LNCaP tumors. Moreover, Wnt3a/α-SMA (*p* = 0.0125) and SDF-1/α-SMA (*p* = 0.0073) co-localization ratios were significantly higher in C4-2B (4.31% ± 2.85% and 20.02% ± 3.30%, respectively) than in LNCaP tumors (Figure 3D). Furthermore, RT-qPCR indicated that the Wnt3a (*p* < 0.0001) and SDF-1 (*p* < 0.0001) mRNA levels were significantly higher in CAFs than in WPMY-1 cells (Figure 3E). Collectively, these results suggest that CAF activation enhanced the tumorigenicity of CRPCs by secreting or locally overexpressing Wnt3a and SDF-1, potentially activating downstream pathways.

### 3.4 CAF-derived Wnt and SDF-1 regulate key pathways in PCSCs

**FIGURE 4:**
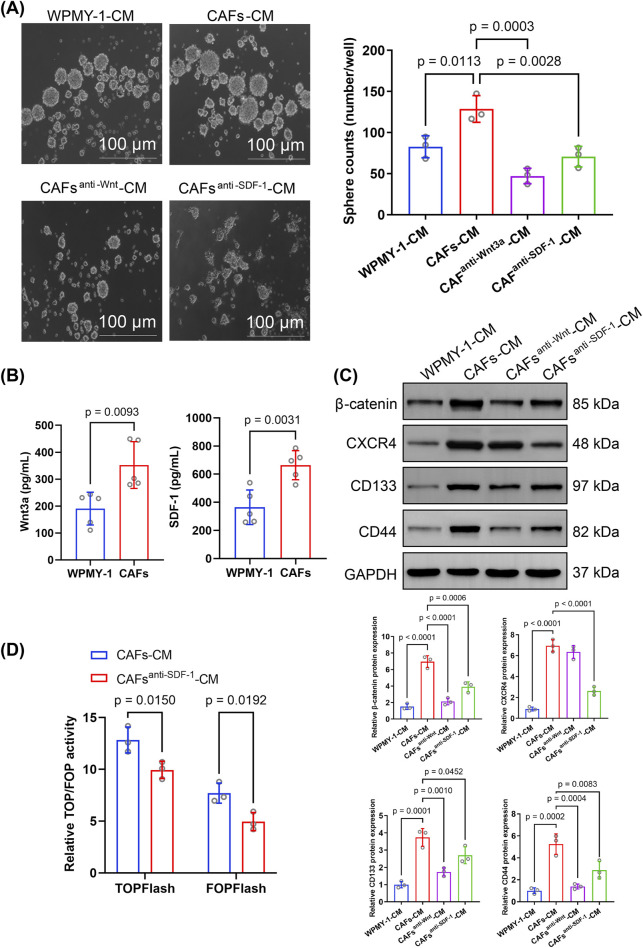
Effect of cancer-associated fibroblast-conditioned medium (CAF-CM) on sphere formation, protein expression, and signaling in prostate cancer stem cells (PCSCs). **(A)** Sphere formation assay of PCSCs cultured in different conditioned media, including WPMY-1-CM, CAF-CM, CAF-CM with the Wnt inhibitor DKK-1 (CAF^anti−Wnt3a^-CM), and CAF-CM with an anti-SDF-1 monoclonal antibody (CAF^anti−SDF-1^-CM). CAF-CM significantly increased sphere formation compared to WPMY-1-CM, while both CAF^anti−Wnt3a^-CM and CAF^anti−SDF-1^-CM significantly reduced sphere formation. Quantification was based on three independent experiments, and the results were analyzed using one-way ANOVA followed by Tukey’s *post hoc* test (n = 3 technical replicates). **(B)** ELISA was performed to measure Wnt3a and SDF-1 levels in conditioned media from WPMY-1 and CAFs. CAFs secreted significantly higher levels of Wnt3a and SDF-1 than WPMY-1 cells. Data were analyzed using an unpaired two-tailed *t*-test (n = 3 technical replicates). **(C)** Western blotting for β-catenin, CXCR4, CD133, and CD44 expression in PCSCs exposed to different CM. Quantification was based on three independent protein extractions. Band intensities were normalized to GAPDH expression and analyzed using one-way ANOVA with Tukey’s *post hoc* test (n = 3 technical replicates). **(D)** TOP/FOPFlash reporter gene assay was used to assess Wnt/β-catenin signaling activity in PCSCs. Treatment with CAF^anti−SDF-1^-CM significantly reduced TOPFlash and FOPFlash activities compared to that with CAF-CM. Data were analyzed using an unpaired two-tailed *t*-test (n = 3 technical replicates). Data are presented as mean ± SD.

Microscopic images showed that CAF-CM treatment significantly enhanced the sphere-forming ability of PCSCs compared to WPMY-1-CM (Figure 4A). Western blotting showed the marked upregulation of CD133 and CD44 expression in PCSCs treated with CAF-CM (Figure 4C), suggesting that CAFs enhanced stem cell-like properties in PCa via a paracrine pathway.

The ELISA showed that the Wnt3a and SDF-1 levels were markedly higher in CAF-CM than in WPMY-1-CM (Figure 4B). Upon inhibition of Wnt3a and SDF-1 expression in CAFs-CM, the number of spheres formed by PCSCs was significantly decreased (Figure 4A). Additionally, inhibition of Wnt or SDF-1 expression in CAF-CM significantly downregulated CD133 and CD44 expression in PCSCs (Figure 4C), indicating that CAF-derived Wnt3a and SDF-1 were essential for promoting stem cell-like characteristics in PCSCs.

Notably, CAF-CM treatment markedly upregulated β-catenin and CXCR4 expression in PCSCs. Compared to CAF-CM treatment, CAF^anti−Wnt^-CM treatment significantly reduced β-catenin level in PCSCs, whereas CAF^anti−SDF-1^-CM treatment significantly inhibited both CXCR4 and β-catenin expression (Figure 4C). Considering the strong association between the SDF-1/CXCR4 axis and β-catenin in PCa, this result suggests that CXCR4 enhanced stem cell-like properties in PCa by activating β-catenin.

To test this hypothesis, we used the TOPFlash luciferase assay to evaluate the effect of SDF-1/CXCR4 signaling on β-catenin downstream transcriptional activity. Importantly, TOPFlash and FOPFlash activities were significantly downregulated in PCSCs incubated in CAF-CM treated with an SDF-1 neutralizing antibody (Figure 4D), further supporting the role of SDF-1 in driving Wnt/β-catenin pathway activation in PCSCs. Collectively, these findings suggest that activated CAFs may be crucial in maintaining stemness and promoting CRPC progression by expressing Wnt3a and SDF-1, thereby activating the Wnt/β-catenin and SDF-1/CXCR4 signaling pathways.

### 3.5 3.5 CAFs promote PCSC stemness *in vivo* via the Wnt/β-catenin and SDF-1/CXCR4 signaling pathways

**FIGURE 5:**
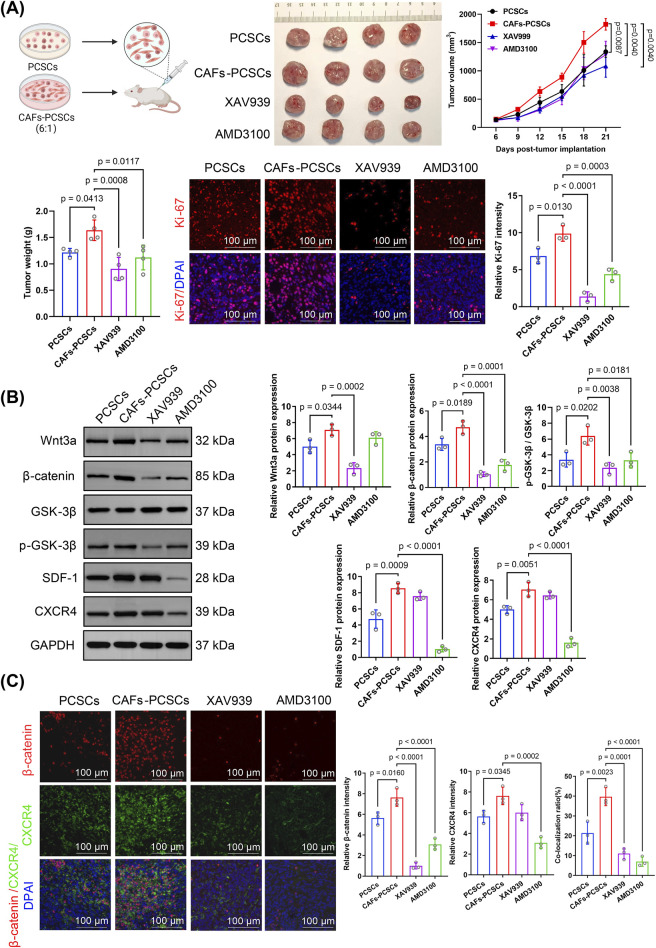
Effects of cancer-associated fibroblasts (CAFs) on prostate cancer stem cell (PCSC) growth and proliferation through Wnt/β-catenin and SDF-1/CXCR4 signaling. **(A)** *In vivo* tumor growth in the PCSC-only and CAF–PCSC groups (1:6 ratio) and treated with or without the Wnt inhibitor XAV939 and the CXCR4 inhibitor AMD3100. Treatment with XAV939 and AMD3100 significantly inhibited tumor growth in the CAF–PCSC group. At the endpoint, tumor weight was significantly higher in the CAF–PCSC group than in the PCSC-only group. In contrast, treatment with XAV939 and AMD3100 significantly decreased tumor weight. Ki-67 immunofluorescence staining (red) was used to assess tumor cell proliferation, and the nuclei were stained with DAPI (blue). The relative intensity of Ki-67 was markedly reduced in the XAV939 and AMD3100 groups, indicating decreased tumor proliferation. **(B)** Protein expression in PCSCs incubated in different conditioned media was analyzed using Western blotting. Wnt3a, β-catenin, SDF-1, and CXCR4 levels and the p-GSK-3β/GSK-3β ratio were significantly higher in the CAF–PCSC group than in the PCSC-only group. XAV939 significantly inhibited Wnt3a and β-catenin expression and the p-GSK-3β/GSK-3β ratio. In contrast, AMD3100 treatment significantly inhibited β-catenin, SDF-1, and CXCR4 expression and the p-GSK-3β/GSK-3β ratio. **(C)** The immunofluorescence assay demonstrated that the CAF–PCSC group had significantly higher β-catenin and CXCR4 fluorescence intensities than the PCSC-only group, with a co-localization rate of 39.71% ± 3.71%. Additionally, XAV939 treatment significantly decreased the relative fluorescence intensity of β-catenin and the co-localization rate of β-catenin and CXCR4 compared with those in the CAF–PCSC group. Similarly, AMD3100 treatment significantly reduced the relative fluorescence intensity of β-catenin and the co-localization rate of β-catenin and CXCR4. The data are presented as mean ± SD (n = 3).

Tumor growth was faster and tumor weight was significantly higher (*p* = 0.00413) in mice co-implanted with CAF–PCSC than in those implanted with PCSCs only. However, treatment with the Wnt inhibitor XAV939 (*p* = 0.0008) and the CXCR4 inhibitor AMD3100 (*p* = 0.0117) significantly reduced tumor weight in the CAF–PCSC co-implantation group (Figure 5A). Immunofluorescence staining showed that Ki-67 expression was significantly higher in the CAF–PCSC group than in the PCSC group, indicating that CAFs enhanced the *in vivo* growth of PCSCs. Similarly, XAV939 and AMD3100 treatments significantly suppressed Ki-67 expression in PCSCs *in vivo* (Figure 5A).

**FIGURE 6:**
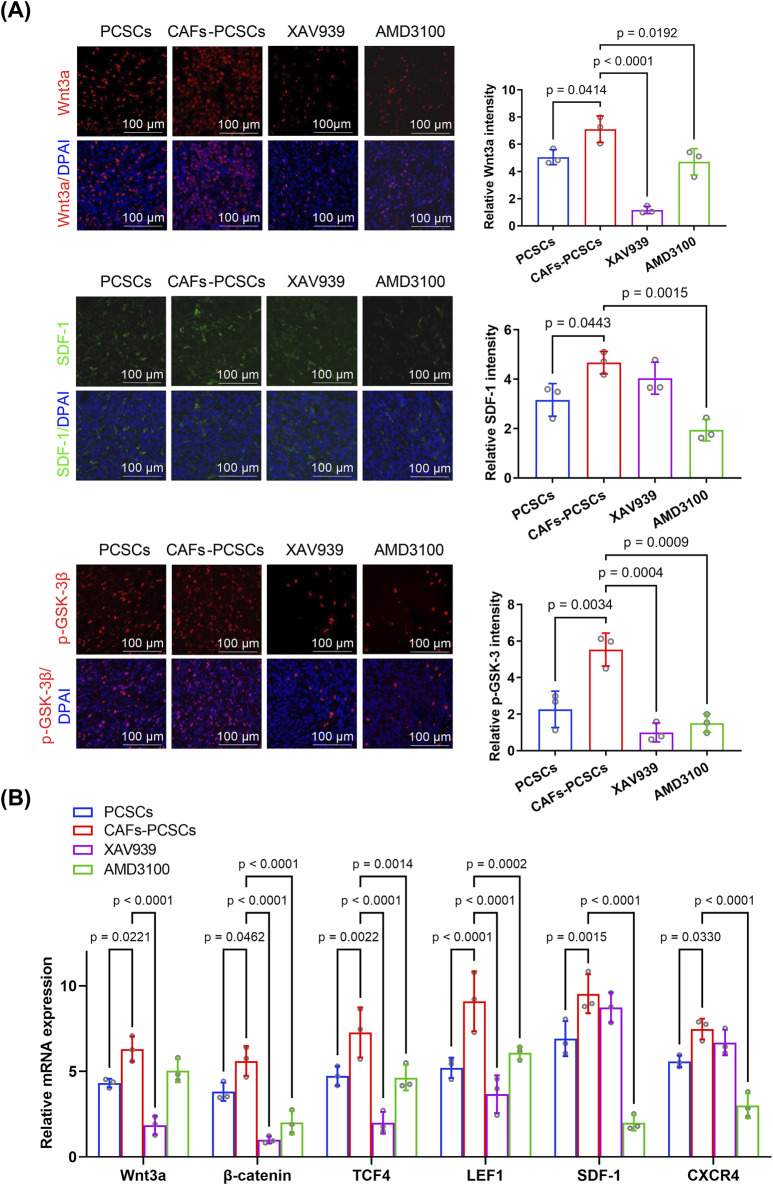
Effects of cancer-associated fibroblasts (CAFs) on prostate cancer stem cell (PCSC) growth and proliferation through the Wnt/β-catenin and SDF-1/CXCR4 signaling pathways. **(A)** Immunofluorescence analysis of PCSCs co-cultured with CAFs (6:1 ratio) and treated with the Wnt inhibitor XAV939 or the CXCR4 inhibitor AMD3100. Wnt3a (red), SDF-1 (green), and p-GSK-3β (red) expression levels were significantly higher in the CAF–PCSC group than in the PCSC-only group. Treatment with XAV939 significantly decreased Wnt3a and p-GSK-3β expression, whereas that with AMD3100 significantly reduced the expression of Wnt3a, SDF-1, and p-GSK-3β. Quantification of fluorescence intensity was performed based on three independent experiments (n = 3 technical replicates), and statistical comparisons were made using one-way ANOVA followed by Tukey’s *post hoc* test. **(B)** RT-qPCR analysis of *Wnt3a*, *β-catenin*, *TCF4*, *LEF1*, *SDF-1*, and *CXCR4* mRNA levels in PCSCs under various treatment conditions. Co-culture with CAFs significantly increased the expression of all six genes compared with that in the PCSC-only group. Treatment with XAV939 significantly reduced *Wnt3a*, *β-catenin*, *TCF4*, and *LEF1* expression, whereas that with AMD3100 significantly reduced *β-catenin*, *TCF4*, *LEF1*, *SDF-1*, and *CXCR4* expression. Data were obtained from three independent experiments (n = 3 technical replicates) and analyzed using one-way ANOVA with Tukey’s *post hoc* test. Data are presented as mean ± SD.

Western blotting showed that Wnt3a, β-catenin, SDF-1, and CXCR4 expression and the p-GSK-3β/GSK-3β ratio were significantly higher in the CAF–PCSC group than in the PCSC group (Figure 5B). RT-qPCR demonstrated that CAF treatment increased the expression of Wnt3a, β-catenin, TCF/LEF (the primary downstream effector of the Wnt pathway), SDF-1, and CXCR4 in PCSCs *in vivo*. Additionally, Wnt3a, β-catenin, p-GSK-3, SDF-1, and CXCR4 fluorescence intensities were higher in the CAF–PCSC group than in the PCSC group (Figure 6A).

Notably, XAV939 treatment suppressed Wnt3a, β-catenin, p-GSK-3, and TCF/LEF expression but did not affect SDF-1 or CXCR4 expression compared with that in the CAF–PCSC group. Additionally, AMD3100 treatment markedly decreased SDF-1, CXCR4, β-catenin, p-GSK-3, and TCF/LEF expression (Figures 5B, 6A,B).

Immunofluorescence staining showed a significant increase in the fluorescence intensities of β-catenin (*p* = 0.0160) and CXCR4 (*p* = 0.0345) in the CAF–PCSC group. Additionally, the co-localization ratio of β-catenin and CXCR4 was significantly higher (*p* = 0.0023) in the CAF–PCSC (39.71% ± 3.71%) group than in the PCSC group (21.32% ± 4.56%; Figure 5C). Moreover, XAV939 and AMD3100 treatments significantly reduced the relative expression and co-localization of β-catenin and CXCR4. XAV939 and AMD3100 treatments significantly decreased the co-localization ratios of β-catenin and CXCR4 to 11.04% ± 2.27% (*p* = 0.0001) and 6.96% ± 1.95% (*p* < 0.0001), respectively.

Based on the results of the *in vitro* and *in vivo* experiments, CXCR4 promoted prostate cancer stem-like properties by activating β-catenin and its downstream pathways. Conclusively, CAFs promoted PCSC proliferation and tumor growth by enhancing the Wnt/β-catenin and SDF-1/CXCR4 signaling pathways. Inhibitors targeting these pathways effectively suppressed the CAF-induced maintenance of cancer stemness.

## 4 Discussion

HSPC progression to CRPC presents a considerable challenge in PCa treatment (Tuxhorn et al., 2002). Notably, this progression involves complex mechanisms associated with various contributing factors, with PCSCs regarded as the primary driving force (Bisson and Prowse, 2009). PCSCs possess self-renewal and multi-lineage differentiation abilities (Chen H. et al., 2024) and can adapt to selective therapeutic pressures through mechanisms such as increased drug efflux, anti-apoptotic gene expression, and active DNA repair (Maitland and Collins, 2008). Importantly, PCa progression to CRPC may be related to paracrine signaling from the stroma, mainly originating from CAFs (Westaby et al., 2022; Banerjee et al., 2023). As an important component of the TME, CAFs regulate PCSC stemness via paracrine pathways and extracellular matrix remodeling (Hou et al., 2024). The heterogeneity and plasticity of PCa, along with the complex interactions between CAFs and PCSCs, pose major challenges for the effective implementation of targeted therapies. In this study, we investigated the mechanisms through which CAF-mediated PCSC proliferation contributes to CRPC progression.

Our findings showed that the CAF-mediated stemness of CSCs is critical for CRPC. The immunohistochemistry and immunofluorescence assays indicated a significant increase in the expression of the CAF markers FAP and α-SMA in the CRPC group. Additionally, the expression of the CSC markers CD133 and CD44 was significantly upregulated in CRPC tumors, further supporting the hypothesis that CAFs promote tumor progression by regulating PCSC properties.

Research indicates that CAF-CM or co-culture with CAF systems can augment cancer cell stemness; stimulate the expression of CD44 and CD133; enhance spheroid formation *in vitro*; and facilitate the self-renewal and proliferation of CSCs in malignancies such as lung (Liu et al., 2022), prostate (Giridharan et al., 2022), breast (Cui et al., 2023), and colorectal cancers (Owen et al., 2022). These pro-stemness effects are mediated through multiple mechanisms. CAFs secrete cytokines such as IL-6, CXCL12, and TGF-β1, which activate oncogenic pathways including the STAT3, Wnt/β-catenin, and CXCR4 pathways to maintain CSC traits (Yang et al., 2023). Moreover, CAF-derived extracellular vesicles, including exosomes and microvesicles, have been shown to deliver functional RNAs and proteins to tumor cells, promoting stemness, therapy resistance, and microenvironmental remodeling (Li et al., 2021). Together, these findings highlight the multifaceted role of CAFs in modulating CSC behavior through both paracrine signaling and vesicle-mediated communication. Giannoni et al. reported that CAFs co-cultured with PCa cells developed a phenotype necessary for the maintenance of tumor stemness following the activation of specific signaling pathways by signals originating from cancer cells or the TME (Su et al., 2018; Li et al., 2020). Similarly, CAFs enhanced the proliferation and spheroid-formation abilities of PCSCs in the present study, with the most pronounced effect observed at a CAF/PCSC ratio of 1:6. Additionally, we generated a murine model via the subcutaneous co-injection of PCSCs and CAFs (1:6) to investigate the *in vivo* effect of CAFs on PCSCs. Notably, CAFs significantly promoted tumor formation *in vivo*. It is widely recognized that PCSCs derived from C4-2B cells exhibit stronger stemness properties than those from LNCaP cells. In this study, rather than re-evaluating these phenotypic differences, we focused on exploring the underlying mechanisms and found that CAF-derived signals play a key role in promoting PCSC enrichment and activity.

CAFs are crucial in sustaining PCSC stemness through the Wnt/β-catenin and SDF-1/CXCR4 signaling pathways. The Wnt/β-catenin pathway is essential for modulating PCSC activity, enhancing DNA repair, and suppressing apoptosis in CSC (Ren et al., 2019). CAF activation induces stemness through Wnt activation, thereby promoting the expression of CSC markers (Liu et al., 2019). Importantly, the binding of Wnt ligands to Frizzled receptors and LRP5/6 co-receptors induces β-catenin dephosphorylation, resulting in β-catenin accumulation and translocation to the nucleus, where it interacts with target genes to activate their expression and regulate CSC activity (Giannoni et al., 2011; Giannoni et al., 2010) reported that Wnt ligands secreted by CAFs can induce CSC characteristics and differentiation resistance in tumor cells. Moreover, the expression of Wnt genes (*Wnt2*, *Wnt3a*, *Wnt7a*, and *Wnt16*) was upregulated in a 3D co-culture of tumor cells and CAFs, with Wnt3a and Wnt16 activating Wnt signaling in both cancer cells and CAFs and promoting the CSC phenotype (Song et al., 2024; Yu et al., 2024) showed that Wnt5a expression was upregulated in SLC14A1^+^ irCAFs and that it positively correlated with the generation of CD133^+^ and CD44^+^CD24^+^ epithelial cells, indicating that Wnt5a from SLC14A1^+^ irCAFs can promote the formation of stem cell-like phenotypes in bladder cancer cells. In addition to the Wnt/β-catenin pathway, the SDF-1/CXCR4 chemokine pathway is essential for modulating PCSCs. CAF-derived SDF-1 activates downstream CXCR4 signaling to promote epithelial–mesenchymal transition, thereby contributing to CSC activity (Stuart et al., 2019). Moreover, the CXCL12γ isoform has been shown to accelerate tumor progression in CRPC by promoting the PCSC phenotype via CXCR4-mediated PKCα/NF-κB activation, resulting in an increase in the population of CD133^+^CD44^+^ CSC-like cells in CXCL12γ-overexpressing tumors (Koushyar et al., 2022). In this study, the ELISA revealed that the levels of the stemness-associated proteins Wnt3a and SDF-1 were significantly higher in CAF-CM than in WPMY-1-CM. Notably, inhibiting the related signaling pathways using the Wnt inhibitor DKK-1 and an SDF-1 neutralizing antibody significantly decreased spheroid formation and the expression of stem cell markers (CD133 and CD44) in PCSCs. Additionally, treatment with β-catenin and CXCR4 inhibitors (XAV939 and AMD3100, respectively) inhibited tumor growth in mice co-injected with CAFs and PCSCs.

Our findings demonstrated that the SDF-1/CXCR4 axis promotes tumor stemness by directly acting on CXCR4, as well as by potentially enhancing PCSC stemness via downstream β-catenin signaling pathway activation. For example, inhibition of CXCL12 activity in CAF-CM significantly decreased TOPFlash luciferase activity in PCSCs. Additionally, inhibition of CXCR4 activity *in vivo* significantly downregulated β-catenin, p-GSK-3β, and TCF/LEF expression, suggesting that the inhibition of CXCR4 activity can effectively suppress GSK-3β phosphorylation and downstream TCF/LEF gene activity, thereby inhibiting Wnt/β-catenin signaling and target gene expression. Importantly, the synergy between these dual signaling pathways (Wnt/β-catenin and SDF-1/CXCR4) has been reported in other cancers, including breast (Hu et al., 2019), bladder (Le et al., 2019), lung (Ma et al., 2022), ovarian (Jung et al., 2018), and colorectal cancers (Zhang et al., 2019); intrahepatic cholangiocarcinoma (Todaro et al., 2014); and osteosarcoma (Wang et al., 2021).

**FIGURE 7:**
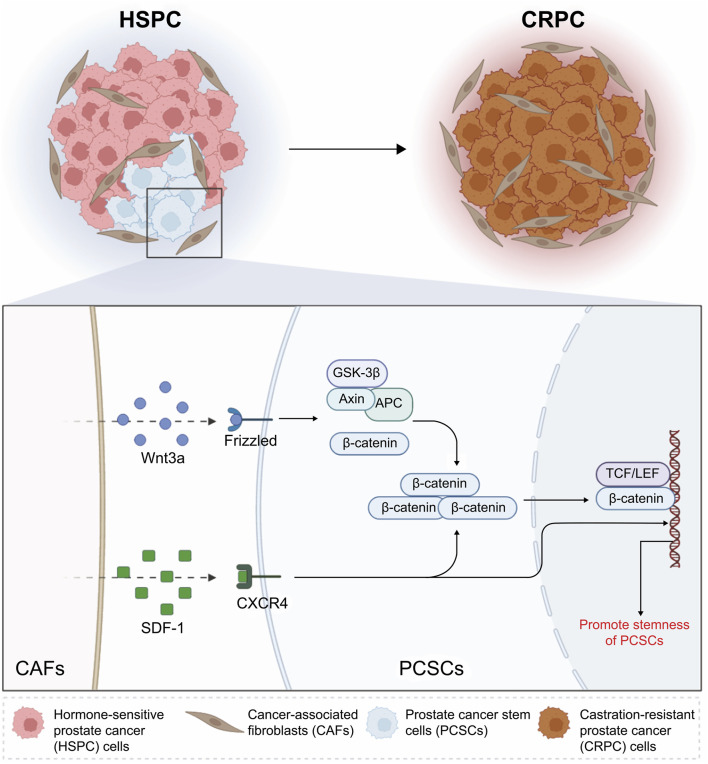
Schematic illustration of the mechanisms through which cancer-associated fibroblasts (CAFs) promote stemness in prostate cancer stem cells (PCSCs) via the Wnt/β-catenin and SDF-1/CXCR4 signaling pathways. In hormone-sensitive prostate cancer, CAFs secrete Wnt3a and SDF-1, which activate the Wnt/β-catenin and SDF-1/CXCR4 pathways, respectively. Wnt3a binds to Frizzled receptors, leading to the stabilization and nuclear translocation of β-catenin. This process promotes the transcription of stemness-related genes through the TCF/LEF complex. Concurrently, SDF-1 interacts with CXCR4 on PCSCs, further enhancing their stemness properties. These interactions contribute to castration-resistant prostate cancer progression by maintaining the stemness and survival of PCSCs. This schematic highlights the critical role of CAFs in regulating PCSC behavior through these signaling pathways. *Created with BioRender.*

Thus, the present study showed that the CAF-mediated paracrine regulation of PCSC stemness is involved in CRPC development and progression. CAFs promote β-catenin accumulation by activating the Wnt/β-catenin and CXCL12/CXCR4 pathways in PCSCs, thereby upregulating the Wnt pathway and promoting PCSC stemness (Figure 7). Our study provides a theoretical basis for the development of targeted therapies against CRPC progression. Moreover, while the activation of Wnt/β-catenin and SDF-1/CXCR4 signaling was supported by molecular evidence, the precise functional consequences of pharmacological inhibition of these pathways in different prostate cancer subtypes and stromal contexts warrant further investigation. These aspects will be important directions for future studies aimed at refining the therapeutic potential of targeting the CAF–PCSC axis.

CAFs play an essential role in maintaining PCSC stemness during CRPC progression. They enhance PCSC self-renewal and proliferation through the Wnt/β-catenin and SDF-1/CXCR4 signaling pathways, with the SDF-1/CXCR4 axis synergistically stimulating the Wnt/β-catenin pathway. Future research should explore additional signaling pathways and potential therapeutic combinations targeting CAF–PCSC interactions to further improve treatment efficacy and overcome resistance in CRPC.

## Data Availability

The datasets presented in this study can be found in online repositories. The names of the repository/repositories and accession number(s) can be found below: https://www.ncbi.nlm.nih.gov/, GSE193337 https://www.ncbi.nlm.nih.gov/, GSE32269.
