# Supplementary material for: CAF-mediated regulation of prostate cancer stem cell stemness via the Wnt/β-catenin and SDF-1/CXCR4 pathways in castration-resistant prostate cancer
Source: Front Cell Dev Biol. 2025 Jul 15;13:1617200. doi: 10.3389/fcell.2025.1617200 (PMC12303950; doi:10.3389/fcell.2025.1617200)
Supplement: Supplementary file 1 [file DataSheet1.docx]

Supplementary Material

# 1 Supplementary Methods

## Enzyme-linked Immunosorbent Assay (ELISA)

CAFs-CM and WPMY-1-CM were collected and analyzed for Wnt3a and SDF-1 levels using human Wnt3a and SDF-1 ELISA kits, respectively (eBioscience, San Diego, CA, USA), following the manufacturer’s instructions. After washing, the cells were incubated for 2 h with biotin-labeled anti-Wnt3a and anti-SDF-1 antibodies, and then with a streptavidin–horseradish peroxidase complex for 30 min. Thereafter, tetramethylbenzidine substrate solution was added, and the cells were incubated for 15 min. Finally, the absorbance was measured at 450 nm using a microplate reader (ELx808; BioTek, Winooski, VT, USA).

## TOP-FLASH/FOP-FLASH Luciferase Reporter Assay

PCSCs were cultured in a 48-well plate and transiently transfected with TOPFlash/FOPFlash luciferase reporter plasmids (BioVector, Beijing, China) and a *Renilla* luciferase control plasmid (Lipofectamine 2000, Invitrogen, CA, USA). After 24 h of treatment with CA-CM and CAF^anti-SDF-1^-CM, the assay was performed according to the instructions of the Dual-Luciferase Reporter Assay System (Promega, Madison, WI, USA). PCSCs were lysed using Passive Lysis Buffer, and the lysate was collected. A 20-µL aliquot of the lysate was added to 100 µL of luciferase assay reagent Ⅱ (LARⅡ) and mixed gently, and the fluorescence value (firefly luciferase, F value) was measured. Thereafter, 100 µL of 1× Stop & Glo reagent was added and mixed, and the fluorescence value (*Renilla* luciferase, R value) was measured. Each experiment was repeated three times. The luciferase activity was calculated as follows:

Luciferase activity = (F_treatment group / R_treatment group) / (F_control group / R_control group) × 100.

## Immunofluorescence and Immunohistochemical Assays

Tumor tissue was fixed in 4% paraformaldehyde solution for 24 h, washed with phosphate-buffered saline (PBS) three times, dehydrated, embedded in optimal cutting temperature (OCT) compound, and cut into 5–10-µm frozen sections. Thereafter, the sections were permeabilized with 0.3% Triton X-100 for 10 min. After blocking with 5% bovine serum albumin (BSA) or 10% normal goat serum for 1 h, the sections were incubated overnight with diluted primary antibodies (against Wnt3a, SDF-1, β-catenin, CXCR4, and p-GSK-3β, all at a dilution of 1:200) at 4 °C. The sections were then incubated in the dark with fluorescence-labeled secondary antibody for 1 h. The nuclei were stained with DAPI and mounted with anti-fade mounting medium. Images were observed and captured in the dark using an optical microscope (BX51; OLYMPUS, Tokyo, Japan).

Immunohistochemistry was performed using an EnVision Detection System (DAKO, Glostrup, Denmark). Tissue sections were mounted on glass slides, deparaffinized, rehydrated using a gradient of xylene and ethanol, and subjected to antigen retrieval by heating in a citrate buffer. After blocking with 5% BSA at 20–25 °C to reduce nonspecific binding, the sections were incubated overnight with the specific primary antibody at 4 °C. Thereafter, the sections were washed with PBS and incubated with secondary antibody at room temperature for 30 min. For color development, the sections were stained with DAB for 1 min and 20 s and counterstained with hematoxylin for 2 min. After nuclear staining, dehydration, and air drying, the sections were mounted with Permount and coverslips, analyzed, and photographed under a light microscope (BX51, OLYMPUS).

# 2 Supplementary Tables

**Table S1**. Antibody and reagent information

| **Antibody/Reagents** | **Supplier** | **Catalog Number** | **Dilution** |
| --- | --- | --- | --- |
| FAP antibody | Abcam (Cambridge, MA, USA) | ab314456 | 1:100 |
| SDF-1 antibody | Abcam (Cambridge, MA, USA) | ab155090 | 1:100 |
| Goat Anti-Rabbit IgG H&L | Abcam (Cambridge, MA, USA) | ab150077 | 1:100 |
| α-SMA antibody | Affinity Biosciences (Cincinnati, OH, USA) | AF1032 | 1:200 |
| CD133 antibody | Affinity Biosciences (Cincinnati, OH, USA) | AF5120 | 1:1000 |
| CD44 antibody | Affinity Biosciences (Cincinnati, OH, USA) | DF6392 | 1:1000 |
| Wnt3a antibody | Affinity Biosciences (Cincinnati, OH, USA) | DF6113 | 1:1000 |
| β-catenin antibody | Affinity Biosciences (Cincinnati, OH, USA) | BF8016 | 1:1000 |
| CXCR4 antibody | Affinity Biosciences (Cincinnati, OH, USA) | AF5279 | 1:1000 |
| GSK-3β antibody | Affinity Biosciences (Cincinnati, OH, USA) | AF5016 | 1:1000 |
| p-GSK-3 antibody | Affinity Biosciences (Cincinnati, OH, USA) | AF2016 | 1:1000 |
| GAPDH antibody | Affinity Biosciences (Cincinnati, OH, USA) | AF7021 | 1:3000 |
| DKK-1 (Wnt inhibitor) | MedChemExpress (Monmouth Junction, NJ, USA) | HY-P72968 | 200 ng/mL |
| AMD3100 (CXCR4 inhibitor) | MedChemExpress (Monmouth Junction, NJ, USA) | HY-10046 | 2.5 mg/mL |
| XAV939 (β-catenin inhibitor) | MedChemExpress (Monmouth Junction, NJ, USA) | HY-15147 | 5 mg/mL |
| Human CXCL12/SDF-1 antibody | R&D Systems (Minneapolis, MN, USA) | MAB310-SP | 1000 nmol/L |

**Table S2**. Polymerase chain reaction primer sequence

| **Gene** | **Primer sequence (5′–3′)** | **GenBank** |
| --- | --- | --- |
| *Wnt3a* (mouse) | F: CTCCTCTCGGATACCTCTTAGTG  R: CCAAGGACCACCAGATCGG | [NM_009522.3](https://www.ncbi.nlm.nih.gov/nuccore/NM_009522.3) |
| *Ctnnb1* (β-catenin gene) (mouse) | F: GAAGAGATGCCGGTTTGTTGA  R: GCCCGAAGCTCCATCACTC | [NM_007614.4](https://www.ncbi.nlm.nih.gov/nuccore/NM_007614.4) |
| *Tcf4* (mouse) | F: CAAGCACTGCCGACTACAATA  R: CCAGGCTGATTCATCCCACTG | [NM_013685.2](https://www.ncbi.nlm.nih.gov/nuccore/NM_013685.2) |
| *Lef1* (mouse) | F: TGCCAAATATGAATAACGACCCA  R: GAGAAAAGTGCTCGTCACTGT | [NM_010703.5](https://www.ncbi.nlm.nih.gov/nuccore/NM_010703.5) |
| *Sdf1* (mouse) | F: GTAGTGGACCTTCCCAACTCT  R: GGCTGTTGTCATACTTCTCATGG | [NM_021704.3](https://www.ncbi.nlm.nih.gov/nuccore/NM_021704.3) |
| *Cxcr4* (mouse) | F: GACTGGCATAGTCGGCAATG  R: AGAAGGGGAGTGTGATGACAAA | [NM_009911.4](https://www.ncbi.nlm.nih.gov/nuccore/NM_009911.4) |
| *Gapdh* (mouse) | F: GGAGCGAGATCCCTCCAAAAT  R: GGCTGTTGTCATACTTCTCATGG | [NM_008084.4](https://www.ncbi.nlm.nih.gov/nuccore/NM_008084.4) |

# 3 Supplementary Results


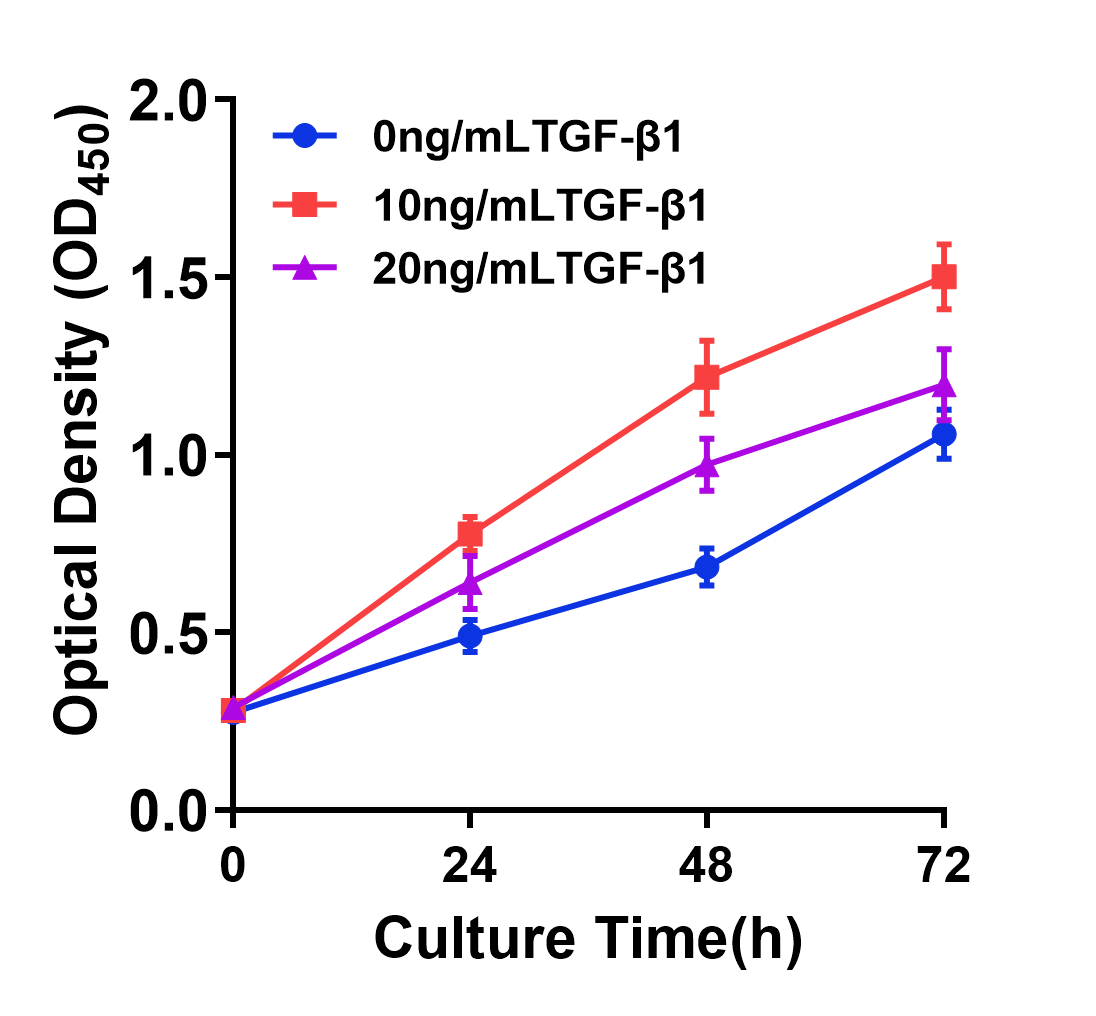


Figure S1. CCK8 assay of WPMY-1 cells treated with TGF-β1.

WPMY-1 cells were treated with 0, 10, or 20 ng/mL TGF-β1 for 24, 48, and 72 h. Cell viability was assessed using the CCK8 assay. Data are presented as mean ± SD (n = 3). Statistical analysis was performed using one-way ANOVA with Tukey’s post hoc test. The results showed that 10 ng/mL TGF-β1 exhibited the optimal activation effect.
